# Redundancy in regulation of lipid accumulation in skeletal muscle during prolonged fasting in obese men

**DOI:** 10.14814/phy2.14285

**Published:** 2019-11-13

**Authors:** Morten L. Høgild, Anders Gudiksen, Henriette Pilegaard, Hans Stødkilde‐Jørgensen, Steen Bønløkke Pedersen, Niels Møller, Jens O. L. Jørgensen, Niels Jessen

**Affiliations:** ^1^ Medical Research Laboratory Department of Clinical Medicine Aarhus University Hospital Aarhus Denmark; ^2^ Department of Clinical Medicine Aarhus University Aarhus Denmark; ^3^ Department of Biology University of Copenhagen Copenhagen Denmark; ^4^ The MR Research Center Aarhus University Hospital Copenhagen Denmark; ^5^ Department of Clinical Pharmacology Aarhus University Hospital Aarhus Denmark; ^6^ Department of Biomedicine Aarhus University Aarhus Denmark

**Keywords:** fasting, Growth hormone, intramyocellular lipid, pyruvate dehydrogenase activity, skeletal muscle

## Abstract

Fasting in human subjects shifts skeletal muscle metabolism toward lipid utilization and accumulation, including intramyocellular lipid (IMCL) deposition. Growth hormone (GH) secretion amplifies during fasting and promotes lipolysis and lipid oxidation, but it is unknown to which degree lipid deposition and metabolism in skeletal muscle during fasting depends on GH action. To test this, we studied nine obese but otherwise healthy men thrice: (a) in the postabsorptive state (“CTRL”), (b) during 72‐hr fasting (“FAST”), and (c) during 72‐hr fasting and treatment with a GH antagonist (GHA) (“FAST + GHA”). IMCL was assessed by magnetic resonance spectroscopy (MRS) and blood samples were drawn for plasma metabolomics assessment while muscle biopsies were obtained for measurements of regulators of substrate metabolism. Prolonged fasting was associated with elevated GH levels and a pronounced GHA‐independent increase in circulating medium‐ and long‐chain fatty acids, glycerol, and ketone bodies indicating increased supply of lipid intermediates to skeletal muscle. Additionally, fasting was associated with a release of short‐, medium‐, and long‐chain acylcarnitines to the circulation from an increased β‐oxidation. This was consistent with a ≈55%–60% decrease in pyruvate dehydrogenase (PDHa) activity. Opposite, IMCL content increased ≈75% with prolonged fasting without an effect of GHA. We suggest that prolonged fasting increases lipid uptake in skeletal muscle and saturates lipid oxidation, both favoring IMCL deposition. This occurs without a detectable effect of GHA on skeletal muscle lipid metabolism.

## Graphical abstract

The present manuscript demonstrates the difference in intramyocellular lipid content in humans during 12‐hr and 48‐hr fasting, respectively. Furthermore, it offers a novel insight into the plasma metabolite profile during 72‐hr fasting. We suggest that intramyocellular lipid content increases during fasting because of supply of lipid intermediates that exceeds lipid oxidation.

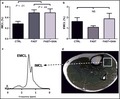

## 1 INTRODUCTION

Skeletal muscle metabolism is a major determinant of overall resting energy expenditure (Zurlo, Larson, Bogardus, & Ravussin, 1990) and is able to rapidly shift between carbohydrate and fat oxidation (Kelley, Goodpaster, Wing, & Simoneau, 1999). It is estimated that skeletal muscle accounts for up to 85% of glucose uptake during an hyper‐insulinemic euglycemic insulin clamp (DeFronzo et al., 1981), whereas only ≈25% of an oral glucose load is disposed in muscle tissue. During prolonged fasting, however, the sensitivity to insulin in skeletal muscle is profoundly reduced (Vendelbo et al., 2012), which increases the availability of glucose for oxidation in other tissues such as the brain. Reduced sensitivity to insulin in skeletal muscle is also a pathophysiological hallmark of type 2 diabetes, and insulin resistance is an early predictor of this disease (Warram, Martin, Krolewski, Soeldner, & Kahn, 1990).

Growth hormone (GH) is an important regulator of substrate metabolism and insulin sensitivity in skeletal muscle (Nellemann et al., 2014). GH treatment reduces overall fat mass (FM) through lipolytic actions in adipose tissues (Salomon, Cuneo, Hesp, & Sonksen, 1989) and decreased adipose tissue triacylglycerol (TAG) synthesis (Rosenbaum, Gertner, & Leibel, 1989). In skeletal muscle, GH shifts substrate metabolism from glucose to lipid oxidation (Nellemann et al., 2014). This is linked to decreased pyruvate dehydrogenase (PDH) activity, which reduces the utilization of glucose intermediates for energy production (Nellemann et al., 2014).

Lipid may accumulate in skeletal muscle if the supply of lipid intermediates exceeds the capacity or demand for its oxidation (Koves et al., 2008). The content of this intramyocellular lipid (IMCL) is associated with insulin resistance in sedentary subjects and type 2 diabetes (Krssak et al., 1999; Pan et al., 1997; Shulman, 2014). IMCL primarily consists of TAGs, which are synthesized from the esterification of fatty acids (FA) and glycerol within skeletal muscle (Bonen, Parolin, et al., 2004; Schwarz, Neese, Turner, Dare, & Hellerstein, 1995). IMCL content depends on continuous FA uptake since de novo lipogenesis is non‐existent in skeletal muscle, wherefore the TAG content in skeletal muscle increases during prolonged fasting with enhanced lipid turnover (Stannard et al., 2002). The uptake of FA for TAG synthesis in myocytes is facilitated by FA transport proteins, binding proteins, and translocase proteins (Glatz, Luiken, & Bonen, 2010). The TAG is stored close to the mitochondria in lipid droplets formed in the endoplasmic reticulum (ER) and these lipid droplets compose a readily available energy source (Hoppeler, 1986; Martin & Parton, 2006; Shaw, Jones, & Wagenmakers, 2008; Thiam, Farese, & Walther, 2013).

In response to an energy demand, TAG is hydrolyzed to FA and glycerol in a process termed lipolysis (Nielsen, Jessen, Jorgensen, Moller, & Lund, 2014). Carnitine‐acylcarnitine translocase (CACT) facilitates transport of FA into the mitochondria for β‐oxidation where carnitine acts as a carrier of FA across the mitochondrial membrane (Hoppel & Genuth, 1980). Acylcarnitines are also transported across the cell membrane via the OCTN2 transporter in a concentration‐dependent manner (Pochini, Oppedisano, & Indiveri, 2004), and an increase in plasma acylcarnitine can indicate incomplete β‐oxidation at a whole‐body level (Hoppel & Genuth, 1980; Koves et al., 2008). Degradation of FA to dicarboxylic acids through ω‐oxidation in the ER only accounts for a small part of FA oxidation during normal conditions, but may constitute a larger fraction during conditions of incomplete or exhaustive β‐oxidation such as fasting (Wanders, Komen, & Kemp, 2011). In addition to its role as a substrate for oxidation, FA are ligands for the peroxisome proliferator‐activated receptor (PPAR) subfamily of transcription factors, which regulate expression of proteins in β‐oxidation (Varga, Czimmerer, & Nagy, 2011).

Several lines of evidence suggest that GH directly regulates skeletal muscle lipid metabolism and IMCL content. Exogenous GH administration to healthy subjects increases IMCL content (Krag et al., 2007), whereas treatment of GH excess with a specific GH antagonist (GHA) reduces IMCL (Madsen et al., 2012). To test the hypothesis that GH regulates lipolysis and IMCL deposition during fasting, we investigated obese but otherwise healthy subjects during prolonged fasting with and without GHA. Obese subjects were chosen because they have higher FA fluxes during fasting compared to lean subjects (Bak et al., 2016). The outcomes were IMCL content, protein levels in muscle of key regulators of lipid metabolism, and relative changes in the plasma metabolome assessed by non‐targeted metabolomics.

## 2 METHODS

### 2.1 Ethical approval

The study was approved by the Central Denmark Region Scientific Ethics Committee (1‐10‐72‐318‐14), conducted in accordance with the Declaration of Helsinki II and reported at http://www.clinicaltrials.gov (NCT02500095). Written consent was obtained from all subjects after appropriate deliberation time prior to oral and written information.

### 2.2 Subjects and study design

Data about study design and subjects characteristics have been published previously (Pedersen et al., 2017). In short, nine healthy overweight and obese men with a median age of 24 years (range: 20–32) and a mean body mass index (BMI) of 30 ± 1.1 kg/m^2^ were included in the study. The subjects were studied thrice separated by at least 4 weeks in a randomized, placebo‐controlled crossover design: (a) During an overnight fast (12 hr) (“CTRL”), (b) during 72‐hr fasting and placebo (“FAST”), and (c) during 72 hr of fasting in combination with GHA (pegvisomant) (“FAST + GHA”). Pegvisomant (SOMAVERT 20 mg; Pfizer, New York City, NY) or placebo (saline) was injected subcutaneously thrice during FAST + GHA: (a) 72 hr prior to initiation of the fasting period, (b) at the beginning of fasting, and (c) after completion of 48 hr of the fasting period. Each study day consisted of a non‐insulin stimulated (“basal”) period from *t* = 0–240 min followed by a 2‐hr hyper‐insulinemic euglycemic clamp (HEC) (*t* = 240–360 min). Insulin was infused at a rate of 40 mU/m^2^ body surface area/min during the HEC (Insulin; ACTRAPID; Novo‐Nordisk, Copenhagen, Denmark), which increased mean [Confidence interval (CI)] insulin levels (pmol/l) to 297 [231;382] (CTRL), 254 [177;364] (FAST) and 350 [267;460] (FAST + GHA) during steady state glucose infusion rates.

### 2.3 Magnetic resonance spectroscopy (^1^H‐MRS)

On all three occasions, skeletal muscle lipid content was measured using ^1^H magnetic resonance spectroscopy (^1^H‐MRS) techniques with a Signa Excite 1.5 Tesla twin‐speed scanner (GE Medical Systems, GE Healthcare, Milwaukee, WI), as previously described (Moller, Stodkilde‐Jorgensen, Jensen, & Jorgensen, 2008). Lipid content was quantified in a 2 × 2 × 2 cm^3^ voxel positioned in the largest cross‐sectional area of the left tibialis anterior muscle and separated into IMCL and extramyocellular lipid (EMCL) content. ^1^H‐MRS was performed during 12‐hr fasting (CTRL) and for practical reasons during 48‐hr fasting instead of 72h fasting (FAST and FAST + GHA). The area of interest was identified using an oblique plane T1‐weighted gradient echo pulse sequence (repetition time (TR) = 600 ms; echo time (TE) = 14 ms; number of excitations (NEX) = 1). ^1^H‐MRS of the skeletal muscle was performed using a water suppressed point‐resolved spectroscopy sequence (TR = 2000 ms; TE = 30 ms; NEX = 8), which generated spectra with a mean ± SE full width at half maximum on 11.3 ± 0.15 Hz. The spectra were analyzed using the LCModel software package (version 6.2; Dr Stephen Provencher) by means of a muscle spectroscopy fitting model. The data processing provided an estimate of the lipid to water ratio in the tissue within the voxel (Provencher, 1993). IMCL and EMCL are presented as the ratio (%) of signal (AU) from lipid to the total signal (lipid + water). The spectrum from FAST + GHA was excluded from the analysis in one subject due to a low quality of the spectrum. The fitted model based on this excluded spectrum had a *SD* of 20, which was well above the overall mean [CI] *SD* of 4.3 [2.9; 5.6].

### 2.4 Muscle tissue and PDHa activity

Muscle tissue biopsies were obtained under sterile conditions using local anesthesia at *t* = 60 and *t* = 270 min. Muscle biopsies were obtained from musculus vastus lateralis of the quadriceps muscle with a Bergström cannula and snap‐frozen in liquid nitrogen. All biopsies were stored at − 80 ˚C. 8–15 mg of muscle tissue was subsequently chipped from each biopsy for PDHa activity analysis. The activity of the active form of PDH (PDHa) was determined in vitro using a radioisotopic assay as previously described (Cederblad, Carlin, Constantin‐Teodosiu, Harper, & Hultman, 1990; Constantin‐Teodosiu, Cederblad, & Hultman, 1991; Putman et al., 1993) after homogenizing of the muscle tissue on ice for 50 s using a micro glass homogenizer (Kontes) and quick freezing the samples (<15 s) in liquid nitrogen.

### 2.5 Metabolomics

Non‐targeted metabolomics was performed in plasma obtained at *t* = 0 min at all three occasions. Plasma samples were analyzed by Metabolon Inc. (Morrisville, NC) as previously described (Evans, DeHaven, Barrett, Mitchell, & Milgram, 2009). Briefly, metabolites were separated using ultrahigh performance liquid chromatography‐tandem mass spectroscopy (UPLC‐MS/MS), which provided information on mass to charge, retention time, and fragment ion spectra. These data were used to detect metabolites via an in‐house chemical library and for quantification of relative changes in metabolites (fold changes).

### 2.6 Western blot analysis

Approximately 5 mg of freeze‐dried muscle tissue was prepared for the western blot analysis using the same protocol and equipment as previously described (Moller et al., 2015). Quantification of stain‐free blots was used to control for equal loading and for total protein normalization as previously reported (Gurtler et al., 2013). Antibodies (Ab) against the following proteins were purchased from Cell Signaling Technology (Danvers, MA; numbers in parentheses indicate catalog numbers): Cytochrom c (rabbit, pAb, 4272), succinate dehydrogenase subunit A (SDHA) (rabbit, mAb, 11998), voltage‐dependent anion channel (VDAC) (rabbit, mAb, 4661), PDH‐E1α (rabbit, mAb, 3205), and from Proteintech (Rosemont, IL): Cluster of differentiation 36 (CD36) (mouse, mAb, 66395‐1‐Ig).

### 2.7 Extraction of RNA and quantitative PCR for mRNA analysis

RNA was extracted using TRIzol (Gibco BRL, Life Technologies, Roskilde, Denmark) and the PCRs were performed in duplicate using LightCycler SYBR Green master mix (Roche Applied Science) in a LightCycler 480 (Roche Applied Science) as previously described (Madsen et al., 2012; Vendelbo et al., 2012). The following primer pairs were designed: fatty acid binding protein 4 (FABP4): GCAGATGACAGGAAAGTCAAGAGC and TCTGCACATGTACCAGGACACC, length = 64 base pair (bp). Carnitine palmitoyl transferase Iα (CPT‐1): TTATCAACAAGCCAGACCCC and TATAATCCCCGTCTCAGGGC, length = 195 bp. CPT‐1β: CTCCTTTCCTTGCTGAGGTG and TCTCGCCTGCAATCATGTAG, length = 177 bp. Angiopoietin‐like 4 (ANGPTL4): TAGTCCACTCTGCCTCTCCC and GAGATGGCCCAGCCAGTT, length = 122 bp. Uncoupling protein 2 (UCP2): CCTCATGACAGATGACCTCC and TGTATCTCGTCTTGACCACG, length = 104 bp. Glucose transporter 4 (GLUT4): CCCCATTCCTTGGTTCATCG and ATAGCCTCCGCAACATACTGG, length = 135 bp. Nuclear respiratory factor 1 (NRF1): TCCATCTATCCGGAAGAGGCAAC and TCTAACGTGGCTCGAAGTTTCCG, length = 60 bp. PPARα: CAGGAAAGGCCAGTAACAATCCAC and CAGCGTCTTCTCAGCCATACAC, length = 74bp. PPARδ: TCCTTCCAGCAGCTACACAGAC and ATCTGCAGTTGGTCCAGCAGTG, length = 69bp. PPARγ: GTGGCCGCAGATTTGAAAGAA and CCATTACGGAGAGATCCACGG, length = 162 bp. Peroxisome proliferator‐activated receptor gamma coactivator 1α (PGC‐1α): TTGAAGAGCGCCGTGTGATT and TGTCTCCATCATCCCGCAGAT, length = 129 bp. Pyruvate dehydrogenase kinase 4 (PDK4): ATTTAAGAATGCAATGCGGGC and GCGGTCAATAATTCTCAGGG, length = 151 bp. The housekeeping gene, β2 microglobulin, was amplified using: GAGGCTATCCAGCGTACTCC and AATGTCGGATGGATGAAACCC, length = 111 bp. The expression levels of the housekeeping gene were similar between all groups and interventions. All primers were from DNA Technology (Risskov, Denmark).

### 2.8 Body composition

Body composition was estimated during fasting at *t* = 0 hr and *t* = 72 hr by means of bioelectrical impedance analysis using Tanita body composition analyzer BC‐418 (Tokyo, Japan).

### 2.9 Statistics

Normal distribution was evaluated by inspection of QQ plots of the residuals from the mixed model, and normally distributed data are presented as mean ± SE. Non‐normally distributed data were logarithmically transformed using the “natural logarithm” and presented as geometric mean ± SE on a logarithmic scale on graphs, as logarithmic data (mean ± SE) on a linear scale on graphs (mRNA data), as (geometric) mean + [CI] or as medians (range). All statistical analyses were made using Stata 13 (College Station, TX). Statistical comparisons between the study days were performed by a mixed effect one‐ or two‐way ANOVA with repeated measures to detect possible main effects and interactions between interventions (CTRL, FAST, and FAST + GHA) and time (non‐insulin stimulation vs. HEC) and adjusted for the random order of interventions. Linear pairwise comparisons were performed *post hoc* to compare differences between interventions. *p* values < .05 were considered statistically significant. Unadjusted values are presented in tables and graphs.

## 3 RESULTS

### 3.1 Fasting reduces lean body mass (LBM) and total body water (TBW) independently of GHA

Fasting was not associated with a significant reduction in FM from *t* = 0 hr to *t* = 72‐hr fasting (ΔFM (kg): 0.18 ± 0.43 [FAST] vs. 0.28 ± 0.27 [FAST + GHA]; *p* = .34) but was associated with a reduction in LBM (ΔLBM (kg) 4.6 ± 0.31 [FAST] vs. 3.9 ± 0.28 [FAST + GHA]; *p* < .01) with no effect of GHA (*p* = .07). TBW was reduced during fasting (ΔTBW (Kg): 3.4 ± 0.23 [FAST] vs. 2.8 ± 0.21 [FAST + GHA]; *p* < .01) without an effect of GHA (*p* = .06). This led to an increased fat percentage (%) (1.1 ± 0.4 [FAST] vs. 0.8 ± 0.25 [FAST + GHA]; *p* < .01) during 72‐hr fasting that was independent of GHA (*p* = .39).

### 3.2 Circulating Free FA (FFA), Ketone bodies, and GH increase and insulin levels decrease during fasting

Plasma concentrations of FFA, 3‐hydroxybutyrate, insulin and GH levels have previously been published (Pedersen et al., 2017) and are summarized in Table 1.

**Table 1:** Plasma levels of hormones and metabolites presented as (geometric) mean [CI] at *t* = 0, 48 and 72‐hr fasting as previous reported (Pedersen et al., 2017)

	0 hr		48 hr		72 hr	
	FAST	FAST+GHA	FAST	FAST+GHA	FAST	FAST+GHA
**FFA** (mmol/l)	0.14 [0.05;0.23]	0.14 [0.09;0.19]	0.67 [0.51;0.83]	0.81 [0.60;1.04]	1.12 [0.90;1.34]	1.28 [1.06;1.50]
**3OHB** (μmol/l)	15.8 [11.4;20.3]	17.1 [8.0; 26.2]	1,192 [748;1636]	1,186 [672;1699]	2,327 [1598;3056]	2,268 [1659;2876]
**GH** (ng/ml)	0.05 [0.03;0.09]	0.05 [0.04;0.06]	2.29 [0.54;9.60]	2.66 [1.19;5.95]	0.56 [0.17;1.84]	0.82 [0.23;3.01]
**Insulin** (pmol/l)	185 [103;334]	161 [97;269]	27 [21;35]	33 [25;44]	31 [22;45]	34 [26;46]

Note

*p* < .05 for the effect of time (fasting) for all variables. *p* > .05 for FAST versus FAST + GHA for all variables. 3‐hydroxybutyrate (3OHB). Free fatty acid (FFA).

### 3.3 CD36 Protein content is unaffected by prolonged fasting and GHA

**Figure 1:**
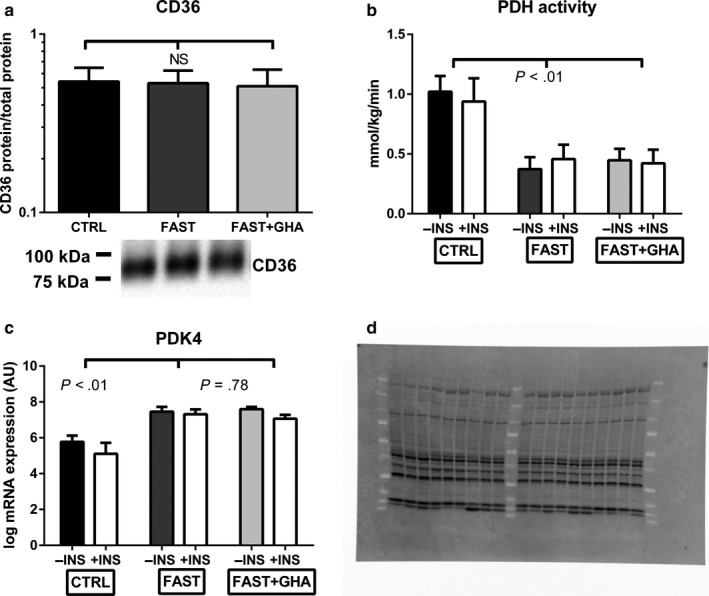
CD36 protein content, PDH activity, and PDK4 expression. (a) Geometric mean ± SE for CD36 protein content. (b) Mean ± SE for PDHa activity. (c) Mean ± SE for log mRNA expression of PDK4. *N* = 9. (d) Stain‐free blot image used for total protein normalization of CD36 (Figure 1a)

Protein expression of CD36 (fatty acids translocase) in skeletal muscle was not influenced by either fasting or GHA (Figure 1a).

### 3.4 PDHa activity decreases during fasting independently of GHA

PDHa activity was measured to quantify the utilization of glucose intermediates (pyruvate) from the glycolytic pathway for oxidation in the citric acid cycle (Putman et al., 1993). The PDHa activity was reduced by ≈ 55%–60% during FAST as well as FAST + GHA compared to CTRL (*p* < .01) without an independent effect of GHA (*p* = .57). Noteworthy, insulin stimulation did not affect PDHa activity during either CTRL, FAST, or FAST + GHA (*p* = .71) (Figure 1b). Reduced PDHa activity was associated with increased PDK4 expression (*p* < .01) without an independent effect of GHA (*p* = .78). There was a small decrease in PDK4 expression during insulin stimulation independent of fasting or GHA (*p* = .46 for the interaction) (Figure 1c).

### 3.5 Fasting increases the mRNA content of PPAR‐regulated target genes involved in FA oxidation

**Figure 2:**
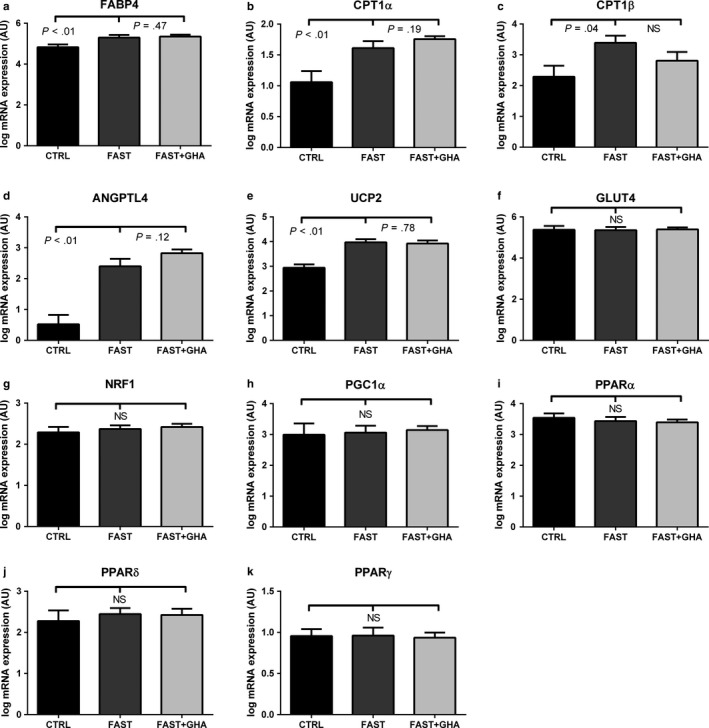
mRNA content of PPAR‐regulated genes and PPARα, β, and γ. (a) Mean ± SE for log mRNA content of FABP4. (b) Mean ± SE for log mRNA content of CPT1α. (c) Mean ± SE for log mRNA content of CPT1α. (d) Mean ± SE for log mRNA content of ANGPTL4. (e) Mean ± SE for log mRNA content of UCP2. (f) Mean ± SE for log mRNA content of GLUT4. (g) Mean ± SE for log mRNA content of NRF1. (h) Mean ± SE for log mRNA content of PGC1α. (i) Mean ± SE for log mRNA content of PPARα. (j) Mean ± SE for log mRNA content of PPARβ. (k) Mean ± SE for log mRNA content of PPARγ. 12‐hr fasting (CTRL), 72‐hr fasting (FAST), and 72‐hr fasting + GHA (FAST + GHA). *N* = 9. Not significant (NS). *N* = 9

Prolonged fasting was associated with a GHA‐independent increase in mRNA content of FABP4, CPT‐1α, ANGPTL4, and UCP2, whereas the mRNA content of CPT‐1β only increased during fasting without GHA (Figure 2a–e). GLUT4 and NRF1 were unaltered by prolonged fasting and GHA (Figure 2f–G). PGC1α, PPARα, PPARδ, and PPARγ mRNA content was also unchanged during prolonged fasting (Figure 2i–K).

### 3.6 IMCL accumulates during prolonged fasting independently of GHA

**Figure 3:**
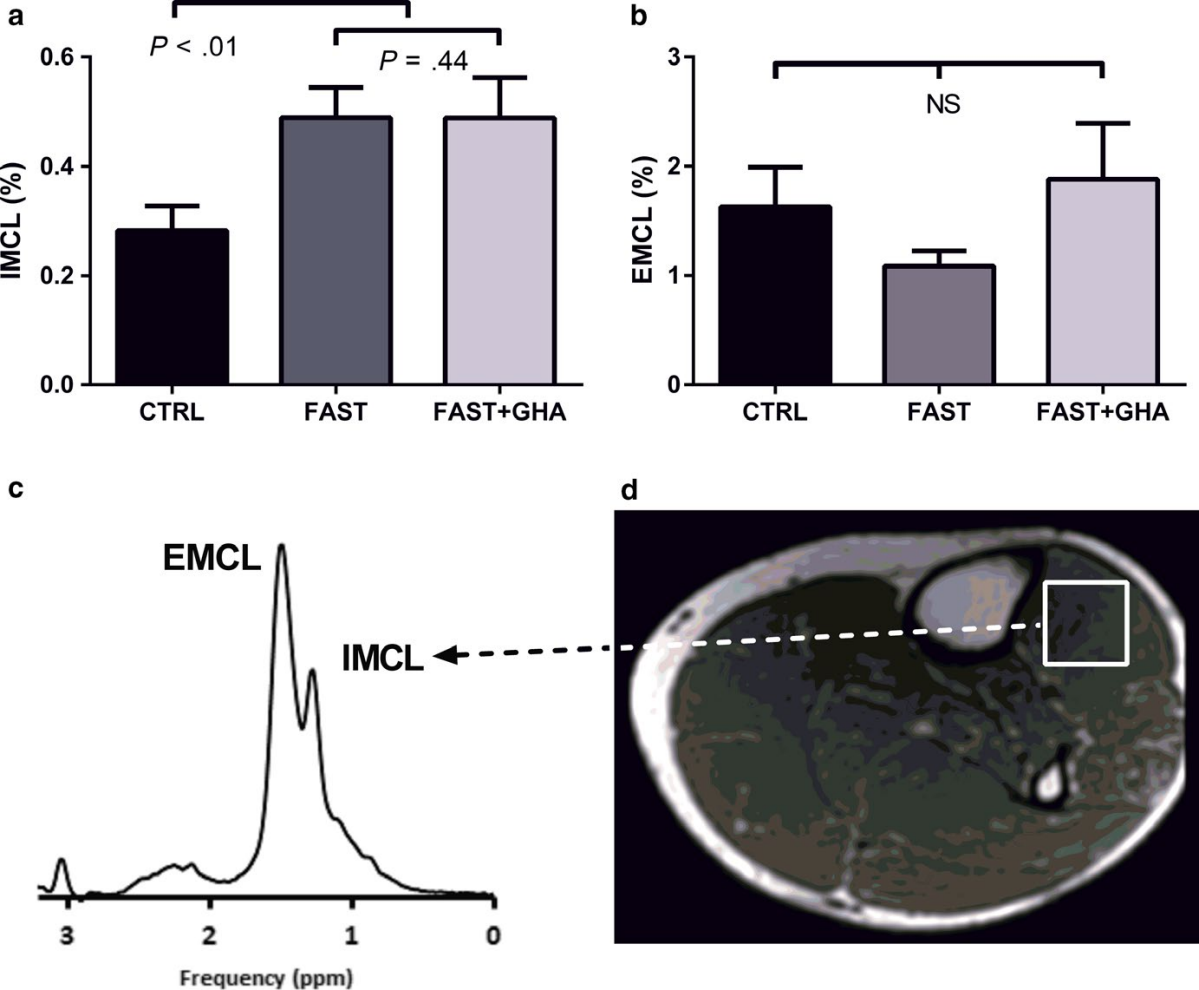
IMCL and EMCL during 72‐hr fasting. (a) Mean ± SE for the relative IMCL content. (b) Mean ± SE for the relative EMCL content. (c) Representative water‐suppressed point‐resolved spectroscopy sequence of the volume of interest in musculus tibialis anterior. (d) Representative image of an oblique‐plane T1‐weighted gradient echo pulse sequences with the voxel positioned in a homogeneous part of the musculus tibialis anterior. 12‐hr fasting (CTRL), 72‐hr fasting (FAST), and 72‐hr fasting + GHA (FAST + GHA). *N* = 8. Not significant (NS).

The relative IMCL content increased ≈75% with FAST and FAST + GHA compared to CTRL (*p* < .01) without an independent effect of GHA (*p* = .44) (Figure 3a). EMCL was unaffected by both fasting and GHR blockade (Figure 3b).

### 3.7 Mitochondrial protein content is unaltered during fasting

**Figure 4:**
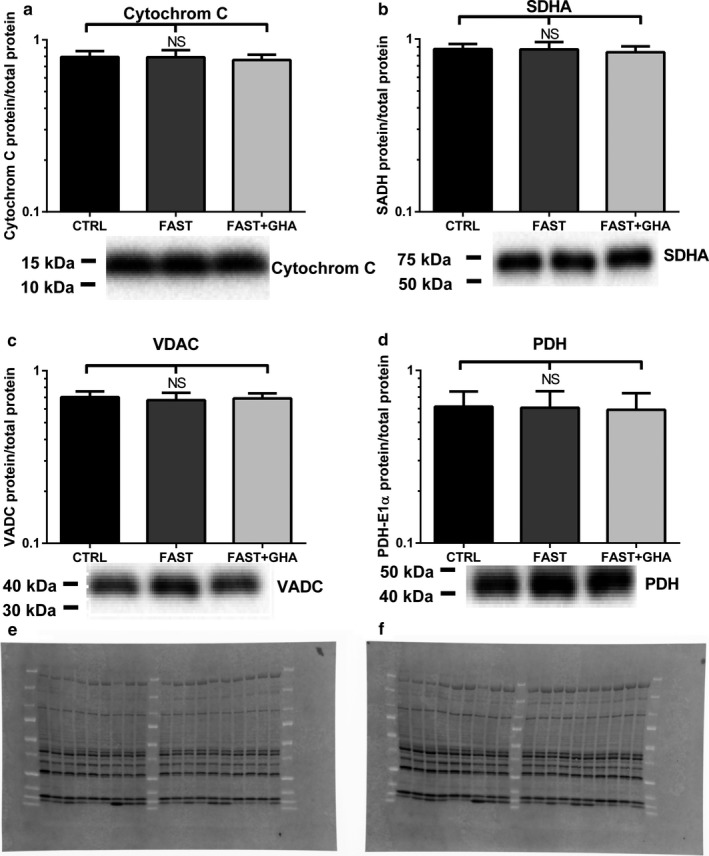
Protein content of mitochondrial proteins (a) Geometric mean ± SE for cytochrome c. (b) Geometric mean ± SE for SDHA. (c) Geometric mean ± SE for VDAC. (d) Geometric mean ± SE for PDH‐E1α. 12‐hr fasting (CTRL), 72‐hr fasting (FAST), and 72‐hr fasting + GHA (FAST + GHA). *N* = 9. Not significant (NS). Representative western blots are for CTRL, FAST, and FAST + GHA are shown below the figures. *N* = 9. (e) Stain‐free blot image used for total protein normalization of Cytochrom C, SDHA, and VDAC (Figure 4a–c). (f) Stain‐free blot image used for total protein normalization of PDH (Figure 4d)

The content of the mitochondrial proteins cytochrome c, SDHA, VDAC, and PDH‐E1α did not change in response to FAST or FAST + GHA (Figure 4a–d).

### 3.8 Prolonged fasting shifts the plasma metabolome from glucose to lipid intermediates

#### 3.8.1 Fasting induces prominent medium‐ and long‐chain fatty acid increase

**Figure 5:**
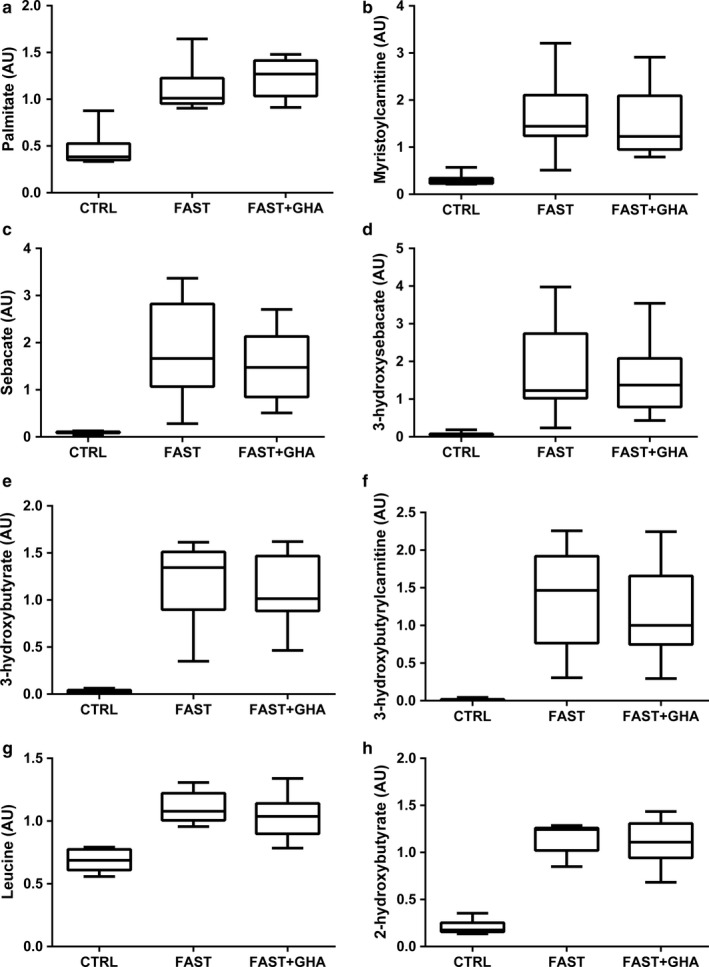
Plasma metabolite concentrations measured at *t* = 0 min during 12‐hr fasting, 72‐hr fasting alone, and 72‐hr fasting with concomitant pegvisomant administration. (a–h) Data for each metabolite are presented as box and whiskers plot (maximum value, 75th percentile, median, 25th percentile, minimum value). 12‐hr fasting (CTRL), 72‐hr fasting (FAST), and 72‐hr fasting + GHA (FAST + GHA). *N* = 9

Fasting was associated with a substantial increase in lipid intermediates (e.g., myristate, arachidate, and linoleate) and a decrease in carbohydrate intermediates (e.g., glucose, fructose, pyruvate, and glycerate) (Table 2). Fasting increased medium‐chain FA ≈1.5 times and induced a tripling of long‐chain FA content (*p* < .05) (Figure 5a and Table 2). The same effect was observed for polyunsaturated and branched‐chain FA (*p* < .05) (Table 2). Monoacylglycerols, diacylglycerols, free glycerol, and sphingolipids also increased (*p* < .05), whereas phospholipids decreased in response to FAST and FAST + GHA (Table 2). There was no overall effect of GHA on plasma FA metabolite distribution besides the short‐chain FA “valerate,” which decreased statistically significant during FAST + GHA (Fold change = 0.42; *p* < .05).

**Table 2:** Selected metabolites during CTRL, FAST, and FAST + GHA

Metabolites		Fold change			Repeated Measures ANOVA					
Group	Specific metabolites	FAST CTRL	FAST + GHA CTRL	FAST + GHA FAST	FAST/CTRL		FAST + GHA/CTRL		FAST + GHA/FAST	
					*p* value	*Q* value	*p* value	*Q* value	*p* value	*Q* value
Carbohydrate	Glucose	0.81	0.84	1.05	.00	0.00	.00	0.00	.21	1.00
	Pyruvate	0.84	0.80	0.95	.09	0.03	.03	0.01	.59	1.00
	Glycerate	0.88	0.81	0.92	.03	0.01	.00	0.00	.16	1.00
	Fructose	0.48	0.47	0.98	.00	0.00	.00	0.00	.79	1.00
	Mannitol/sorbitol	0.33	0.36	1.10	.00	0.00	.01	0.00	.67	1.00
Short‐chain fatty acid	Valerate	0.81	0.42	0.51	.22	0.07	.01	0.00	.11	1.00
Medium‐chain fatty acid	Caprylate	1.48	1.66	1.12	.00	0.00	.00	0.00	.29	1.00
	Caprate	1.64	1.53	0.93	.00	0.00	.00	0.00	.40	1.00
	Laurate	1.80	1.98	1.10	.00	0.00	.00	0.00	.35	1.00
	5‐Dodecenoate	2.99	3.37	1.13	.00	0.00	.00	0.00	.74	1.00
Long‐chain fatty acid	Myristate	2.51	2.93	1.17	.00	0.00	.00	0.00	.23	1.00
	Myristoleate	2.26	2.55	1.13	.00	0.00	.00	0.00	.59	1.00
	Pentadecanoate	2.06	2.34	1.14	.00	0.00	.00	0.00	.24	1.00
	Palmitate	2.45	2.71	1.11	.00	0.00	.00	0.00	.31	1.00
	Palmitoleate	2.60	3.23	1.24	.00	0.00	.00	0.00	.28	1.00
	Margarate	3.06	3.34	1.09	.00	0.00	.00	0.00	.50	1.00
	Stearate	2.23	2.27	1.02	.00	0.00	.00	0.00	.86	1.00
	Arachidate	2.12	2.32	1.09	.00	0.00	.00	0.00	.66	1.00
	Eicosenoate	3.55	3.83	1.08	.00	0.00	.00	0.00	.59	1.00
	Oleate/vaccenate	2.60	2.80	1.08	.00	0.00	.00	0.00	.51	1.00
Polyunsaturated fatty acid (n3 and n6)	Stearidonate	1.24	1.57	1.26	.11	0.04	.01	0.00	.25	1.00
	Eicosapentaenoate	1.81	2.03	1.13	.00	0.00	.00	0.00	.60	1.00
	Docosapentaenoate	2.50	2.82	1.13	.00	0.00	.00	0.00	.39	1.00
	Docosahexaenoate	1.81	1.93	1.07	.00	0.00	.00	0.00	.66	1.00
	Linoleate	2.56	2.88	1.12	.00	0.00	.00	0.00	.29	1.00
	Arachidonate	1.91	2.07	1.09	.00	0.00	.00	0.00	.41	1.00
	Adrenate	2.72	3.01	1.10	.00	0.00	.00	0.00	.39	1.00
Fatty acid, branched	13‐Methylmyristate	1.94	2.15	1.11	.00	0.00	.00	0.00	.65	1.00
	15‐Methylpalmitate	2.27	2.40	1.06	.00	0.00	.00	0.00	.72	1.00
	17‐Methylstearate	2.29	1.89	0.83	.00	0.00	.00	0.00	.04	1.00
Fatty acid, dicarboxylate	Adipate	9.09	7.76	0.85	.00	0.00	.00	0.00	.43	1.00
	Sebacate (decanedioate)	19.79	15.96	0.81	.00	0.00	.00	0.00	.62	1.00
	Dodecanedioate	16.59	14.08	0.85	.00	0.00	.00	0.00	.83	1.00
	Tetradecanedioate	12.05	10.68	0.89	.00	0.00	.00	0.00	.90	1.00
	Hexadecanedioate	10.09	9.26	0.92	.00	0.00	.00	0.00	.84	1.00
	Octadecanedioate	4.49	3.89	0.87	.00	0.00	.00	0.00	.54	1.00
Fatty acid, amino	2‐Aminoheptanoate	0.39	0.47	1.22	.00	0.00	.00	0.00	.03	1.00
	2‐Aminooctanoate	0.61	0.64	1.05	.01	0.00	.02	0.01	.64	1.00
Fatty acid synthesis	Malonate	1.20	1.58	1.31	.43	0.12	.02	0.01	.08	1.00
Fatty acid metabolism (also BCAA metabolism)	Butyrylcarnitine	1.23	1.22	0.99	.02	0.01	.03	0.01	.82	1.00
	Butyrylglycine	1.51	1.53	1.01	.04	0.02	.04	0.01	.97	1.00
	Propionylcarnitine	0.73	0.66	0.91	.00	0.00	.00	0.00	.17	1.00
	Propionylglycine	0.24	0.23	0.96	.00	0.00	.00	0.00	.85	1.00
	Methylmalonate	0.55	0.57	1.04	.00	0.00	.00	0.00	.55	1.00
Fatty acid metabolism (Acyl Glycine)	Hexanoylglycine	2.26	2.19	0.97	.00	0.00	.00	0.00	.67	1.00
	*N*‐Palmitoylglycine	1.58	1.50	0.94	.00	0.00	.00	0.00	.77	1.00
Fatty acid metabolism (Acyl Carnitine)	Acetylcarnitine	3.79	3.39	0.89	.00	0.00	.00	0.00	.24	1.00
	3‐Hydroxybutyrylcarnitine	73.64	63.05	0.86	.00	0.00	.00	0.00	.56	1.00
	Hexanoylcarnitine	2.64	2.68	1.01	.00	0.00	.00	0.00	.99	1.00
	Octanoylcarnitine	2.40	2.50	1.04	.00	0.00	.00	0.00	1.00	1.00
	Decanoylcarnitine	2.56	2.70	1.05	.00	0.00	.00	0.00	.79	1.00
	Laurylcarnitine	4.31	3.94	0.92	.00	0.00	.00	0.00	.73	1.00
	Myristoylcarnitine	5.46	4.90	0.90	.00	0.00	.00	0.00	.49	1.00
	Palmitoylcarnitine	2.16	2.12	0.98	.00	0.00	.00	0.00	.72	1.00
	Palmitoleoylcarnitine	4.95	4.78	0.97	.00	0.00	.00	0.00	.95	1.00
	Linolenoylcarnitine	2.42	2.66	1.10	.00	0.00	.00	0.00	.36	1.00
	Oleoylcarnitine	2.36	2.45	1.04	.00	0.00	.00	0.00	.51	1.00
	Myristoleoylcarnitine	6.63	6.68	1.01	.00	0.00	.00	0.00	.88	1.00
	Suberoylcarnitine	9.47	8.52	0.90	.00	0.00	.00	0.00	.36	1.00
	Docosahexaenoylcarnitine	1.94	1.59	0.82	.00	0.00	.01	0.00	.18	1.00
	Margaroylcarnitine	2.26	2.01	0.89	.00	0.00	.00	0.00	.33	1.00
Carnitine metabolism	Deoxycarnitine	0.98	1.00	1.02	.74	0.19	.82	0.20	.92	1.00
	Carnitine	0.78	0.77	0.99	.00	0.00	.00	0.00	.85	1.00
Ketone bodies	Acetoacetate	30.35	31.84	1.05	.00	0.00	.00	0.00	.78	1.00
	3‐Hydroxybutyrate	44.97	42.03	0.93	.00	0.00	.00	0.00	.91	1.00
Fatty acid, monohydroxy	3‐Hydroxyhexanoate	5.54	5.25	0.95	.00	0.00	.00	0.00	.75	1.00
	3‐Hydroxyoctanoate	5.63	5.26	0.93	.00	0.00	.00	0.00	.98	1.00
	3‐Hydroxydecanoate	4.03	4.22	1.05	.00	0.00	.00	0.00	.92	1.00
	3‐Hydroxysebacate	25.04	21.48	0.86	.00	0.00	.00	0.00	.72	1.00
Fatty acid, dihydroxy	12.13‐Dihome	0.77	0.85	1.12	.04	0.01	.14	0.04	.48	1.00
	9.10‐Dihome	1.57	1.62	1.03	.01	0.00	.00	0.00	.77	1.00
Monoacylglycerol	1‐Linoleoylglycerol	1.27	1.30	1.02	.13	0.04	.06	0.02	.67	1.00
	1‐Arachidonylglycerol	1.36	2.17	1.60	.01	0.01	.00	0.00	.05	1.00
	1‐Docosahexaenoylglycerol	1.55	1.96	1.27	.02	0.01	.00	0.00	.27	1.00
Diacylglycerol	Linoleoyl‐arachidonoyl‐glycerol	2.84	2.95	1.04	.00	0.00	.00	0.00	.94	1.00
	Linoleoyl‐docosahexaenoyl‐glycerol	4.44	3.52	0.79	.00	0.00	.00	0.00	.25	1.00
	Palmitoyl‐arachidonoyl‐	5.25	6.47	1.23	.00	0.00	.00	0.00	.34	1.00
	Palmitoyl‐docosahexaenoyl‐glycerol	4.88	4.16	0.85	00	0.00	.00	0.00	.31	1.00
Glycerolipid metabolism	Glycerol	2.15	3.09	1.44	.00	0.00	.00	0.00	.06	1.00
	Glycerol 3‐phosphate	1.06	0.93	0.88	.68	0.18	.48	0.13	.27	1.00
Phospholipid metabolism	1‐Palmitoleoyl−2‐linolenoyl‐GPC	0.23	0.23	0.97	.00	0.00	.00	0.00	.57	1.00
	1.2‐Dilinoleoyl‐GPC	0.26	0.26	0.99	.00	0.00	.00	0.00	.95	1.00
	Sphingomyelin	3.04	3.05	1.00	.00	0.00	.00	0.00	.83	1.00
Fatty acid metabolism (Acyl Choline)	Palmitoylcholine	0.60	0.77	1.29	.09	0.03	.37	0.11	.38	1.00
	Oleoylcholine	0.51	0.62	1.21	.04	0.01	.12	0.04	.54	1.00
	Linoleoylcholine	0.41	0.52	1.27	.01	0.00	.05	0.02	.38	1.00
	Stearoylcholine	0.36	0.46	1.27	.02	0.01	.07	0.02	.59	1.00
Fatty acid metabolism (Acyl Glutamine)	Hexanoylglutamine	22.62	21.93	0.97	.00	0.00	.00	0.00	.79	1.00
Leucine, isoleucine and valine metabolism	Leucine	1.60	1.50	0.94	.00	0.00	.00	0.00	.15	1.00
	Isovalerate	1.53	1.51	0.99	.00	0.00	.00	0.00	.88	1.00
	Isovalerylglycine	1.49	1.36	0.91	.04	0.01	.11	0.04	.59	1.00
	Beta‐hydroxyisovalerate	1.61	1.90	1.18	.00	0.00	.00	0.00	.30	1.00
	alpha‐hydroxyisovalerate	3.09	3.34	1.08	.00	0.00	.00	0.00	.65	1.00
	Isoleucine	1.67	1.63	0.98	.00	0.00	.00	0.00	.52	1.00
	2‐Methylbutyrylcarnitine	1.39	1.23	0.89	.02	0.01	.27	0.08	.20	1.00
	2‐Hydroxy−3‐methylvalerate	3.38	3.51	1.04	.00	0.00	.00	0.00	1.00	1.00
	3‐Hydroxy−2‐ethylpropionate	2.24	2.35	1.05	.00	0.00	.00	0.00	.75	1.00
	Valine	1.39	1.31	0.94	.00	0.00	.00	0.00	.16	1.00
Methionine, cysteine, and taurine metabolism	Methionine	0.91	0.88	0.97	.12	0.04	.05	0.02	.65	1.00
	Methionine sulfone	1.33	1.44	1.08	.02	0.01	.00	0.00	.52	1.00
	Methionine sulfoxide	0.78	0.79	1.01	.00	0.00	.00	0.00	1.00	1.00
	Cystathionine	0.73	0.64	0.88	.12	0.04	.10	0.03	.89	1.00
	Alpha‐ketobutyrate	3.57	3.32	0.93	.00	0.00	.00	0.00	.53	1.00
	2‐Aminobutyrate	2.44	2.36	0.96	.00	0.00	.00	0.00	.47	1.00
	Cysteine	1.24	1.00	0.81	.06	0.02	.99	0.24	.06	1.00
	Cystine	1.13	1.10	0.98	.52	0.14	.50	0.14	.98	1.00
	S‐Methylcysteine	0.95	1.07	1.12	.95	0.23	.37	0.11	.40	1.00
	Taurine	1.27	1.27	1.00	.03	0.01	.03	0.01	.93	1.00
	2‐Hydroxybutyrate/2‐hydroxyisobutyrate	5.57	5.37	0.96	0.00	0.00	.00	0.00	.66	1.00
Threonine	Threonine	0.69	0.68	0.99	.00	0.00	.00	0.00	.86	1.00

Note

Fold changes (FAST vs. CTRL, FAST + GHA vs. CTRL, and FAST + GHA vs. FAST) of specific metabolites grouped into different pathways of the metabolism are provide together with the P values and Q values of the repeated measures ANOVA. Branched‐chain amino acids (BCAA). *N* = 9

#### 3.8.2 Fasting is associated with elevated levels of intermediates from β‐oxidation and ω‐oxidation

The majority of short‐, medium‐, and long‐chain acylcarnitines increased significantly (Fold change ≈ 1.5–8) during FAST and FAST + GHA (*p* < .05) indicative of incomplete β‐oxidation (Figure 5b and Table 2). This was accompanied by a decrease in free plasma carnitine concentrations (*p* < .05) (Table 2). FAST was also associated with a 20‐fold increase in the dicarboxylic acid “sebacate” derived from ω‐oxidation, and the hydroxydicarboxylic acid metabolite “3‐hydroxy sebactate,” supporting the concept of ω‐oxidation as a rescue pathway during saturated β‐oxidation (*p* < .05) (Figure 5c–d and Table 2). As expected, FAST also induced a substantial elevation in ketone body levels with a ≈30‐fold and ≈45‐fold increase in circulating acetoacetate and 3‐hydroxybutyrate, respectively (*p* < .05) (Figure 5e and Table 2). The lipid metabolite “3‐hydroxybutyryl‐carnitine” also increased pronouncedly during FAST independent of GHA (*p* < .05) (Figure 5f).

### 3.9 Branched‐chain amino acids (BCAA) and sulfur amino acid (SUAA) metabolites increase during fasting

The three BCAA leucine, isoleucine, and valine together with their downstream metabolites increased during FAST (*p* < .05) without a significant effect of GHA (Figure 5g and Table 2). Prolonged fasting was also associated with GHA‐independent accumulation of several methionine intermediates in plasma together with increased levels of α‐ketobutyrate and its downstream metabolites 2‐aminobutyrate and 2‐hydroxybutyrate (Figure 5h and Table 2).

## 4 DISCUSSION

This study was designed to assess the importance of endogenous GH actions on the intramyocellular lipid content and key regulators of lipid metabolism during prolonged fasting in obese but otherwise healthy subjects. We did not record significant effects of GHA on either of these outcomes, which indicates that redundant mechanisms promote adipose tissue lipolysis and lipid storage in muscle during prolonged fasting. However, these data do not challenge the important role of GH as a regulator of lipid metabolism that has been established in studies of GH‐deficient subjects (Norrelund, Djurhuus, et al., 2003) and in fasting healthy subjects infused with GH (Norrelund, Nair, et al., 2003).

The marked increase in circulating medium‐ and long‐chain FA, monoacylglycerols, diacylglycerols, and free glycerol during prolonged fasting demonstrates an increased supply of lipid intermediates to skeletal muscles. This was not associated with increased protein content of the fatty acid transporter, CD36, in skeletal muscle. This does not preclude increased transport of FAs into muscle, since we were only able to measure total CD36 content rather than the active proportion of the protein, which is translocated to the cell surface (Bonen, Campbell, et al., 2004). PDH is a key regulator of carbohydrate oxidation, and reduced PDHa activity partitions lipid intermediates toward oxidation at the expense of carbohydrates (Randle, Garland, Hales, & Newsholme, 1963). In accordance, prolonged fasting was associated with decreased PDHa activity, which was not modulated by GHA. In lean subjects, administration of exogenous GH reduces PDHa activity in skeletal muscle (Nellemann et al., 2014). This is likely a consequence of increased lipid oxidation and the associated elevated acetyl‐CoA/CoA‐ratio (Putman et al., 1993). In addition, PDHa activity is negatively regulated by pyruvate dehydrogenase kinase 4 (PDK4) (Pilegaard & Neufer, 2004) which is increased by elevated FA levels (Kelley, Mokan, Simoneau, & Mandarino, 1993; Wu, Inskeep, Bowker‐Kinley, Popov, & Harris, 1999). In accordance, we observed increased PDK4 expression during fasting. We did not detected increased PDHa activity during insulin stimulation, but a small decrease in PDK4 expression. This contrasts observations in lean subjects (Mandarino et al., 1987). The underlying mechanism is likely higher levels of PDK4 in muscle from obese versus lean subjects, and the inability of insulin to stimulate PDHa activity suggests that insulin resistance in obese individuals can be detected also at the level of regulators of substrate metabolism. Surprisingly, increased lipolysis and lipid oxidation did not result in significant lower FM measured by bioelectrical impedance. This could be explained by reduced energy expenditure during fasting. However, this study was not powered to detect a difference in body composition using bioelectrical impedance.

The expression of PPARγ target genes such as FABP 4 (Desvergne & Wahli, 1999), ANGPTL4 (Rogue, Spire, Brun, Claude, & Guillouzo, 2010) as well as UCP2 (Desvergne & Wahli, 1999) and CPT‐Iα (Desvergne & Wahli, 1999) increased during prolonged fasting as previously reported (Rosenbaum et al., 1989; Salomon et al., 1989), which was independent of GHA. Noteworthy, CPT‐Iβ expression (the muscle isoform) was unchanged during FAST + GHA compared to CTRL, which indicate hampered FA oxidation during fasting in the presence of GHR blockade. FABP4 controls intracellular lipid transport to lipid droplets and to the mitochondria. CPT‐I is the rate‐limiting enzyme of FA oxidation, as CPT‐I enables influx of long‐chain FA to the mitochondria, and ANGPTL4 is associated with increased lipid mobilization and FA oxidation (Mandard et al., 2006). Increased expression of UCP2 has been linked to protection of mitochondria during fatty acid β‐oxidation (Azzu, Jastroch, Divakaruni, & Brand, 2010; Patterson, Shah, Matsubara, Krausz, & Gonzalez, 2012). Lipid intermediates can act as activators of PPAR‐regulated gene transcription (Varga et al., 2011), and this mechanism is therefore likely to mediate the transcriptional effects independently of canonical GHR‐signaling. Thus, the combined activation of PPAR‐regulated genes increases the capacity for lipid oxidation (Varga et al., 2011) and may play a dominant role in the metabolic adaptation to fasting in muscle tissue. The increases in PPAR‐regulated genes are compatible with transcriptional regulation of β‐oxidation in skeletal muscle. In contrast, we did not find evidence of altered PPARα, β or γ expression. Likewise, we observed unaltered PPARγ coactivator 1‐α (PGC‐1α) activity during prolonged fasting in obese subjects as both the PGC‐1α, GLUT4,and NRF1 mRNA content (Finck & Kelly, 2006) and the protein content of several mitochondrial proteins were preserved.

We chose to study obese but otherwise healthy subjects due to their higher FA fluxes during fasting as compared to lean subjects (Bak et al., 2016). The physiological response to prolonged fasting involves IMCL accumulation in lean subjects, and the present results demonstrate that this also occurs to the same degree in obese subjects. In contrast, the decrease in insulin sensitivity during 72 hr fasting is more pronounced in lean subjects (Pedersen et al., 2017; Vendelbo et al., 2012). Of note, previous studies have shown that IMCL starts to accumulate during ≈24‐hr fasting and continues until at least 120‐hr fasting in lean (Vendelbo et al., 2012; Wietek et al., 2004). Accumulation of IMCL therefore appears to be a physiological adaption to fasting independent of body composition and without direct correlation to the level of insulin resistance. In that sense, it is in accordance with observations of increased IMCL levels in endurance‐trained athletes with high insulin sensitivity (Goodpaster, He, Watkins, & Kelley, 2001). This suggests that the increase in IMCL during prolonged fasting per se is not directly linked to insulin resistance. The present observation that there is no effect of prolonged fasting on EMCL in obese but otherwise healthy subjects is in agreement with data in lean subjects (Stannard et al., 2002). EMCL constitutes a quantitatively larger lipid pool than IMCL for which reason a longer fasting period may be required to induce detectable changes.

The increased availability of lipid intermediates during prolonged fasting provides substrates for ketogenesis, which is in accordance with the observation that plasma levels of ketone bodies increased by >30‐fold in our study. This lipolytic and ketogenic effect can be stimulated by exogenous GH administration during fasting in GH‐deficient patients (Norrelund, Djurhuus, et al., 2003), but in this study of obese but otherwise healthy subjects, the increase in lipid intermediates and ketone bodies was independent of GHA. This discrepancy could be due to the fact that obesity is associated with a blunted elevation in GH secretion and action in response to fasting (Riedel, Hoeft, Blum, von zur Muhlen, & Brabant, 1995). It is also possible that the magnitude of GH blockade, which we obtained, was incomplete as regards lipid metabolism, even though GHA in the present study had a significant impact on serum IGF‐I levels [194.6 ng/ml (saline) vs. 162.6 ng/ml (GHA)] and endogenous glucose production (Pedersen et al., 2017). Indeed, we did record sporadic STAT5 phosphorylation in muscle during GHA in the present study (Pedersen et al., 2017) which could represent GH signaling. One could speculate that bio‐distribution of pegvisomant reduces the accessibility to the receptors in muscle and adipose tissue compared to liver tissue, which could explain the hepatic effects of GHA in the presence of only minor effects in muscle and adipose tissue.

Ketone bodies can serve as substrates for oxidation in skeletal muscle (Randle et al., 1963). The metabolomics analysis performed in this study was conducted as a non‐targeted investigation and we were therefore only able to detect relative changes in metabolites. This limits firm conclusions, but the pronounced increase in circulating “3‐hydroxybutyryl‐carnitine” is compatible with ketone body metabolism in skeletal muscle (Soeters et al., 2012). Our subjects did not perform physical activities during the fasting period, and fasting per se does not lead to energy deprivation in muscle (Vendelbo et al., 2012; Wijngaarden, Zon, Dijk, Pijl, & Guigas, 2013). The observed increases in “3‐hydroxybutyryl‐carnitine” and other acylcarnitines suggest that energy supply exceeds the resting energy requirements during prolonged fasting. Our findings are in accordance with data from metabolomics on skeletal muscle during prolonged fasting (Bak et al., 2018). In humans, the maximal capacity for β‐oxidation can be reached during intravenous lipid infusion in human subjects (Gormsen et al., 2007). ω‐oxidation constitutes a rescue pathway for FA oxidation during exhaustive lipid catabolism (Wanders et al., 2011) and the observed increase in dicarboxylic acids and their downstream metabolites, which can be generated during ω‐oxidation, provide further support for saturated β‐oxidation during prolonged fasting. Yet, the concept of saturated β‐oxidation remains speculative. Acylcarnitines and “3‐hydroxybutyryl‐carnitine” are associated with insulin resistance, and they may act in synergy with BCAA to suppress insulin actions (Newgard, 2012). We demonstrated that acylcarnitines and BCAA also increase in relation to fasting‐induced insulin resistance. Our observations of IMCL accumulation and ω‐oxidation indicate that plasma acylcarnitines increase because of excess energy availability from lipolysis.

In conclusion, fasting induces a selective use of lipids as fuel in skeletal muscle. The massive supply of lipid intermediates is partitioned to lipid oxidation and this may saturate the capacity for β‐oxidation. Lipids also accumulate in skeletal muscle during fasting, which is not modified significantly by GHA, which could indicate either insufficient GH blockade or the existence of redundant mechanisms to ensure lipid mobilization. This also suggests that lipid accumulation in skeletal muscle during fasting is driven by an increased supply that exceeds the demand for lipid utilization in obese subjects. New studies are needed to establish the role of GH on skeletal muscle lipid metabolism during fasting and to clarify the concept of saturable β‐oxidation.

## CONFLICT OF INTEREST

The authors have nothing to disclose.

## References

[phy214285-bib-0001] Azzu, V. , Jastroch, M. , Divakaruni, A. S. , & Brand, M. D. (2010). The regulation and turnover of mitochondrial uncoupling proteins. Biochimica Et Biophysica Acta, 1797, 785–791. 20211596 10.1016/j.bbabio.2010.02.035 PMC2891119

[phy214285-bib-0002] Bak, A. M. , Moller, A. B. , Vendelbo, M. H. , Nielsen, T. S. , Viggers, R. , Rungby, J. , … Moller, N. (2016). Differential regulation of lipid and protein metabolism in obese vs. lean subjects before and after a 72‐h fast. American Journal of Physiology Endocrinology and Metabolism, 311, E224–235. 27245338 10.1152/ajpendo.00464.2015

[phy214285-bib-0003] Bak, A. M. , Vendelbo, M. H. , Christensen, B. , Viggers, R. , Bibby, B. M. , Rungby, J. , … Jessen, N. (2018). Prolonged fasting‐induced metabolic signatures in human skeletal muscle of lean and obese men. PLoS ONE, 13, e0200817. 30183740 10.1371/journal.pone.0200817 PMC6124727

[phy214285-bib-0004] Bonen, A. , Campbell, S. E. , Benton, C. R. , Chabowski, A. , Coort, S. L. , Han, X. X. , … Luiken, J. J. (2004). Regulation of fatty acid transport by fatty acid translocase/CD36. The Proceedings of the Nutrition Society, 63, 245–249. 15294038 10.1079/PNS2004331

[phy214285-bib-0005] Bonen, A. , Parolin, M. L. , Steinberg, G. R. , Calles‐Escandon, J. , Tandon, N. N. , Glatz, J. F. , … Dyck, D. J. (2004). Triacylglycerol accumulation in human obesity and type 2 diabetes is associated with increased rates of skeletal muscle fatty acid transport and increased sarcolemmal FAT/CD36. FASEB Journal : Official Publication of the Federation of American Societies for Experimental Biology, 18, 1144–1146. 15132977 10.1096/fj.03-1065fje

[phy214285-bib-0006] Cederblad, G. , Carlin, J. I. , Constantin‐Teodosiu, D. , Harper, P. , & Hultman, E. (1990). Radioisotopic assays of CoASH and carnitine and their acetylated forms in human skeletal muscle. Analytical Biochemistry, 185, 274–278. 2339783 10.1016/0003-2697(90)90292-h

[phy214285-bib-0007] Constantin‐Teodosiu, D. , Cederblad, G. , & Hultman, E. (1991). A sensitive radioisotopic assay of pyruvate dehydrogenase complex in human muscle tissue. Analytical Biochemistry, 198, 347–351. 1799221 10.1016/0003-2697(91)90437-x

[phy214285-bib-0008] DeFronzo, R. A. , Jacot, E. , Jequier, E. , Maeder, E. , Wahren, J. & Felber, J. P. (1981). The effect of insulin on the disposal of intravenous glucose. Results from indirect calorimetry and hepatic and femoral venous catheterization. Diabetes, 30, 1000–1007. 7030826 10.2337/diab.30.12.1000

[phy214285-bib-0009] Desvergne, B. , & Wahli, W. (1999). Peroxisome proliferator‐activated receptors: Nuclear control of metabolism. Endocrine Reviews, 20, 649–688. 10529898 10.1210/edrv.20.5.0380

[phy214285-bib-0010] Evans, A. M. , DeHaven, C. D. , Barrett, T. , Mitchell, M. , & Milgram, E. (2009). Integrated, nontargeted ultrahigh performance liquid chromatography/electrospray ionization tandem mass spectrometry platform for the identification and relative quantification of the small‐molecule complement of biological systems. Analytical Chemistry, 81, 6656–6667. 19624122 10.1021/ac901536h

[phy214285-bib-0011] Finck, B. N. , & Kelly, D. P. (2006). PGC‐1 coactivators: Inducible regulators of energy metabolism in health and disease. The Journal of Clinical Investigation, 116, 615–622. 16511594 10.1172/JCI27794 PMC1386111

[phy214285-bib-0012] Glatz, J. F. , Luiken, J. J. , & Bonen, A. (2010). Membrane fatty acid transporters as regulators of lipid metabolism: Implications for metabolic disease. Physiological Reviews, 90, 367–417. 20086080 10.1152/physrev.00003.2009

[phy214285-bib-0013] Goodpaster, B. H. , He, J. , Watkins, S. , & Kelley, D. E. (2001). Skeletal muscle lipid content and insulin resistance: Evidence for a paradox in endurance‐trained athletes. The Journal of Clinical Endocrinology and Metabolism, 86, 5755–5761. 11739435 10.1210/jcem.86.12.8075

[phy214285-bib-0014] Gormsen, L. C. , Jessen, N. , Gjedsted, J. , Gjedde, S. , Norrelund, H. , Lund, S. , … Moller, N. (2007). Dose‐response effects of free fatty acids on glucose and lipid metabolism during somatostatin blockade of growth hormone and insulin in humans. The Journal of Clinical Endocrinology and Metabolism, 92, 1834–1842. 17341555 10.1210/jc.2006-2659

[phy214285-bib-0015] Gurtler, A. , Kunz, N. , Gomolka, M. , Hornhardt, S. , Friedl, A. A. , McDonald, K. , … Posch, A. (2013). Stain‐Free technology as a normalization tool in Western blot analysis. Analytical Biochemistry, 433, 105–111. 23085117 10.1016/j.ab.2012.10.010

[phy214285-bib-0016] Hoppel, C. L. , & Genuth, S. M. (1980). Carnitine metabolism in normal‐weight and obese human subjects during fasting. The American Journal of Physiology, 238, E409–415. 7377339 10.1152/ajpendo.1980.238.5.E409

[phy214285-bib-0017] Hoppeler, H. (1986). Exercise‐induced ultrastructural changes in skeletal muscle. International Journal of Sports Medicine, 7, 187–204. 3531039 10.1055/s-2008-1025758

[phy214285-bib-0018] Kelley, D. E. , Goodpaster, B. , Wing, R. R. , & Simoneau, J. A. (1999). Skeletal muscle fatty acid metabolism in association with insulin resistance, obesity, and weight loss. The American Journal of Physiology, 277, E1130–1141. 10600804 10.1152/ajpendo.1999.277.6.E1130

[phy214285-bib-0019] Kelley, D. E. , Mokan, M. , Simoneau, J. A. , & Mandarino, L. J. (1993). Interaction between glucose and free fatty acid metabolism in human skeletal muscle. The Journal of Clinical Investigation, 92, 91–98. 8326021 10.1172/JCI116603 PMC293539

[phy214285-bib-0020] Koves, T. R. , Ussher, J. R. , Noland, R. C. , Slentz, D. , Mosedale, M. , Ilkayeva, O. , … Muoio, D. M. (2008). Mitochondrial overload and incomplete fatty acid oxidation contribute to skeletal muscle insulin resistance. Cell Metabolism, 7, 45–56. 18177724 10.1016/j.cmet.2007.10.013

[phy214285-bib-0021] Krag, M. B. , Gormsen, L. C. , Guo, Z. , Christiansen, J. S. , Jensen, M. D. , Nielsen, S. , & Jorgensen, J. O. (2007). Growth hormone‐induced insulin resistance is associated with increased intramyocellular triglyceride content but unaltered VLDL‐triglyceride kinetics. American Journal of Physiology Endocrinology and Metabolism, 292, E920–927. 17132823 10.1152/ajpendo.00374.2006

[phy214285-bib-0022] Krssak, M. , Falk Petersen, K. , Dresner, A. , DiPietro, L. , Vogel, S. M. , Rothman, D. L. , … Shulman, G. I. (1999). Intramyocellular lipid concentrations are correlated with insulin sensitivity in humans: A 1H NMR spectroscopy study. Diabetologia, 42, 113–116. 10027589 10.1007/s001250051123

[phy214285-bib-0023] Madsen, M. , Krusenstjerna‐Hafstrom, T. , Moller, L. , Christensen, B. , Vendelbo, M. H. , Pedersen, S. B. , … Jorgensen, J. O. (2012). Fat content in liver and skeletal muscle changes in a reciprocal manner in patients with acromegaly during combination therapy with a somatostatin analog and a GH receptor antagonist: A randomized clinical trial. The Journal of Clinical Endocrinology and Metabolism, 97, 1227–1235. 22298804 10.1210/jc.2011-2681

[phy214285-bib-0024] Mandard, S. , Zandbergen, F. , van Straten, E. , Wahli, W. , Kuipers, F. , Muller, M. , & Kersten, S. (2006). The fasting‐induced adipose factor/angiopoietin‐like protein 4 is physically associated with lipoproteins and governs plasma lipid levels and adiposity. The Journal of Biological Chemistry, 281, 934–944. 16272564 10.1074/jbc.M506519200

[phy214285-bib-0025] Mandarino, L. J. , Wright, K. S. , Verity, L. S. , Nichols, J. , Bell, J. M. , Kolterman, O. G. & Beck‐Nielsen, H. (1987). Effects of insulin infusion on human skeletal muscle pyruvate dehydrogenase, phosphofructokinase, and glycogen synthase. Evidence for their role in oxidative and nonoxidative glucose metabolism. The Journal of Clinical Investigation, 80, 655–663. 2957389 10.1172/JCI113118 PMC442287

[phy214285-bib-0026] Martin, S. , & Parton, R. G. (2006). Lipid droplets: A unified view of a dynamic organelle. Nature Reviews Molecular Cell Biology, 7, 373–378. 16550215 10.1038/nrm1912

[phy214285-bib-0027] Moller, A. B. , Vendelbo, M. H. , Christensen, B. , Clasen, B. F. , Bak, A. M. , Jorgensen, J. O. , … Jessen, N. (2015). Physical exercise increases autophagic signaling through ULK1 in human skeletal muscle. Journal of Applied Physiology (Bethesda, Md: 1985), 118, 971–979. 10.1152/japplphysiol.01116.2014 25678702

[phy214285-bib-0028] Moller, L. , Stodkilde‐Jorgensen, H. , Jensen, F. T. , & Jorgensen, J. O. (2008). Fasting in healthy subjects is associated with intrahepatic accumulation of lipids as assessed by 1H‐magnetic resonance spectroscopy. Clinical Science (London, England: 1979), 114, 547–552. 10.1042/CS20070217 17990983

[phy214285-bib-0029] Nellemann, B. , Vendelbo, M. H. , Nielsen, T. S. , Bak, A. M. , Hogild, M. , Pedersen, S. B. , … Jorgensen, J. O. (2014). Growth hormone‐induced insulin resistance in human subjects involves reduced pyruvate dehydrogenase activity. Acta Physiologica (Oxford, England), 210, 392–402. 10.1111/apha.12183 24148194

[phy214285-bib-0030] Newgard, C. B. (2012). Interplay between lipids and branched‐chain amino acids in development of insulin resistance. Cell Metabolism, 15, 606–614. 22560213 10.1016/j.cmet.2012.01.024 PMC3695706

[phy214285-bib-0031] Nielsen, T. S. , Jessen, N. , Jorgensen, J. O. , Moller, N. , & Lund, S. (2014). Dissecting adipose tissue lipolysis: Molecular regulation and implications for metabolic disease. Journal of Molecular Endocrinology, 52, R199–222. 24577718 10.1530/JME-13-0277

[phy214285-bib-0032] Norrelund, H. , Djurhuus, C. , Jorgensen, J. O. , Nielsen, S. , Nair, K. S. , Schmitz, O. , … Moller, N. (2003). Effects of GH on urea, glucose and lipid metabolism, and insulin sensitivity during fasting in GH‐deficient patients. American Journal of Physiology Endocrinology and Metabolism, 285, E737–743. 12799313 10.1152/ajpendo.00092.2003

[phy214285-bib-0033] Norrelund, H. , Nair, K. S. , Nielsen, S. , Frystyk, J. , Ivarsen, P. , Jorgensen, J. O. , … Moller, N. (2003). The decisive role of free fatty acids for protein conservation during fasting in humans with and without growth hormone. The Journal of Clinical Endocrinology and Metabolism, 88, 4371–4378. 12970312 10.1210/jc.2003-030267

[phy214285-bib-0034] Pan, D. A. , Lillioja, S. , Kriketos, A. D. , Milner, M. R. , Baur, L. A. , Bogardus, C. , … Storlien, L. H. (1997). Skeletal muscle triglyceride levels are inversely related to insulin action. Diabetes, 46, 983–988. 9166669 10.2337/diab.46.6.983

[phy214285-bib-0035] Patterson, A. D. , Shah, Y. M. , Matsubara, T. , Krausz, K. W. , & Gonzalez, F. J. (2012). Peroxisome proliferator‐activated receptor alpha induction of uncoupling protein 2 protects against acetaminophen‐induced liver toxicity. Hepatology (Baltimore, MD), 56, 281–290. 10.1002/hep.25645 PMC3378765 22318764

[phy214285-bib-0036] Pedersen, M. H. , Svart, M. V. , Lebeck, J. , Bidlingmaier, M. , Stodkilde‐Jorgensen, H. , Pedersen, S. B. , … Jorgensen, J. O. (2017). Substrate Metabolism and Insulin Sensitivity During Fasting in Obese Human Subjects: Impact of GH Blockade. . The Journal of Clinical Endocrinology and Metabolism, 102, 1340–1349. 28324055 10.1210/jc.2016-3835

[phy214285-bib-0037] Pilegaard, H. , & Neufer, P. D. (2004). Transcriptional regulation of pyruvate dehydrogenase kinase 4 in skeletal muscle during and after exercise. The Proceedings of the Nutrition Society, 63, 221–226. 15294034 10.1079/pns2004345

[phy214285-bib-0038] Pochini, L. , Oppedisano, F. , & Indiveri, C. (2004). Reconstitution into liposomes and functional characterization of the carnitine transporter from renal cell plasma membrane. Biochimica Et Biophysica Acta, 1661, 78–86. 14967477 10.1016/j.bbamem.2003.12.001

[phy214285-bib-0039] Provencher, S. W. (1993). Estimation of metabolite concentrations from localized in vivo proton NMR spectra. Magnetic Resonance in Medicine, 30, 672–679. 8139448 10.1002/mrm.1910300604

[phy214285-bib-0040] Putman, C. T. , Spriet, L. L. , Hultman, E. , Lindinger, M. I. , Lands, L. C. , McKelvie, R. S. , … Heigenhauser, G. J. (1993). Pyruvate dehydrogenase activity and acetyl group accumulation during exercise after different diets. The American Journal of Physiology, 265, E752–760. 8238502 10.1152/ajpendo.1993.265.5.E752

[phy214285-bib-0041] Randle, P. J. , Garland, P. B. , Hales, C. N. & Newsholme, E. A. (1963). The glucose fatty‐acid cycle. Its role in insulin sensitivity and the metabolic disturbances of diabetes mellitus. Lancet (London, England), 1, 785–789. 10.1016/s0140-6736(63)91500-9 13990765

[phy214285-bib-0042] Riedel, M. , Hoeft, B. , & Blum, W. F. , von zur Muhlen A. , & Brabant G. (1995). Pulsatile growth hormone secretion in normal‐weight and obese men: Differential metabolic regulation during energy restriction. Metabolism: Clinical and Experimental, 44, 605–610. 7752908 10.1016/0026-0495(95)90117-5

[phy214285-bib-0043] Rogue, A. , Spire, C. , Brun, M. , Claude, N. , & Guillouzo, A. (2010). Gene Expression Changes Induced by PPAR Gamma Agonists in Animal and Human Liver. PPAR Research, 2010, 325183. 20981297 10.1155/2010/325183 PMC2963138

[phy214285-bib-0044] Rosenbaum, M. , Gertner, J. M. , & Leibel, R. L. (1989). Effects of systemic growth hormone (GH) administration on regional adipose tissue distribution and metabolism in GH‐deficient children. The Journal of Clinical Endocrinology and Metabolism, 69, 1274–1281. 2685009 10.1210/jcem-69-6-1274

[phy214285-bib-0045] Salomon, F. , Cuneo, R. C. , Hesp, R. , & Sonksen, P. H. (1989). The effects of treatment with recombinant human growth hormone on body composition and metabolism in adults with growth hormone deficiency. The New England Journal of Medicine, 321, 1797–1803. 2687691 10.1056/NEJM198912283212605

[phy214285-bib-0046] Schwarz, J. M. , Neese, R. A. , Turner, S. , Dare, D. & Hellerstein, M. K. (1995). Short‐term alterations in carbohydrate energy intake in humans. Striking effects on hepatic glucose production, de novo lipogenesis, lipolysis, and whole‐body fuel selection. The Journal of Clinical Investigation, 96, 2735–2743. 8675642 10.1172/JCI118342 PMC185982

[phy214285-bib-0047] Shaw, C. S. , Jones, D. A. , & Wagenmakers, A. J. (2008). Network distribution of mitochondria and lipid droplets in human muscle fibres. Histochemistry and Cell Biology, 129, 65–72. 17938948 10.1007/s00418-007-0349-8

[phy214285-bib-0048] Shulman, G. I. (2014). Ectopic fat in insulin resistance, dyslipidemia, and cardiometabolic disease. The New England Journal of Medicine, 371, 1131–1141. 25229917 10.1056/NEJMra1011035

[phy214285-bib-0049] Soeters, M. R. , Serlie, M. J. , Sauerwein, H. P. , Duran, M. , Ruiter, J. P. , Kulik, W. , … Houten, S. M. (2012). Characterization of D‐3‐hydroxybutyrylcarnitine (ketocarnitine): An identified ketosis‐induced metabolite. Metabolism: Clinical and Experimental, 61, 966–973. 22209095 10.1016/j.metabol.2011.11.009

[phy214285-bib-0050] Stannard, S. R. , Thompson, M. W. , Fairbairn, K. , Huard, B. , Sachinwalla, T. , & Thompson, C. H. (2002). Fasting for 72 h increases intramyocellular lipid content in nondiabetic, physically fit men. American Journal of Physiology Endocrinology and Metabolism, 283, E1185–1191. 12388154 10.1152/ajpendo.00108.2002

[phy214285-bib-0051] Thiam, A. R. , Farese, R. V. Jr , & Walther, T. C. (2013). The biophysics and cell biology of lipid droplets. Nature Reviews Molecular Cell Biology, 14, 775–786. 24220094 10.1038/nrm3699 PMC4526153

[phy214285-bib-0052] Varga, T. , Czimmerer, Z. , & Nagy, L. (2011). PPARs are a unique set of fatty acid regulated transcription factors controlling both lipid metabolism and inflammation. Biochimica Et Biophysica Acta, 1007–1022. 10.1016/j.bbadis.2011.02.014 PMC3117990 21382489

[phy214285-bib-0053] Vendelbo, M. H. , Clasen, B. F. , Treebak, J. T. , Moller, L. , Krusenstjerna‐Hafstrom, T. , Madsen, M. , … Jessen, N. (2012). Insulin resistance after a 72‐h fast is associated with impaired AS160 phosphorylation and accumulation of lipid and glycogen in human skeletal muscle. American Journal of Physiology Endocrinology and Metabolism, 302, E190–200. 22028408 10.1152/ajpendo.00207.2011 PMC4971894

[phy214285-bib-0054] Wanders, R. J. , Komen, J. , & Kemp, S. (2011). Fatty acid omega‐oxidation as a rescue pathway for fatty acid oxidation disorders in humans. The FEBS Journal, 278, 182–194. 21156023 10.1111/j.1742-4658.2010.07947.x

[phy214285-bib-0055] Warram, J. H. , Martin, B. C. , Krolewski, A. S. , Soeldner, J. S. , & Kahn, C. R. (1990). Slow glucose removal rate and hyperinsulinemia precede the development of type II diabetes in the offspring of diabetic parents. Annals of Internal Medicine, 113, 909–915. 2240915 10.7326/0003-4819-113-12-909

[phy214285-bib-0056] Wietek, B. M. , Machann, J. , Mader, I. , Thamer, C. , Haring, H. U. , Claussen, C. D. , … Schick, F. (2004). Muscle type dependent increase in intramyocellular lipids during prolonged fasting of human subjects: A proton MRS study. *Hormone and metabolic research =* . Hormon‐ Und Stoffwechselforschung = Hormones Et Metabolisme, 36, 639–644. 15486816 10.1055/s-2004-825928

[phy214285-bib-0057] Wijngaarden, M. A. , van der Zon, G. C. , van Dijk, K. W. , Pijl, H. , & Guigas, B. (2013). Effects of prolonged fasting on AMPK signaling, gene expression, and mitochondrial respiratory chain content in skeletal muscle from lean and obese individuals. American Journal of Physiology Endocrinology and Metabolism, 304, E1012–1021. 23512807 10.1152/ajpendo.00008.2013

[phy214285-bib-0058] Wu, P. , Inskeep, K. , Bowker‐Kinley, M. M. , Popov, K. M. , & Harris, R. A. (1999). Mechanism responsible for inactivation of skeletal muscle pyruvate dehydrogenase complex in starvation and diabetes. Diabetes, 48, 1593–1599. 10426378 10.2337/diabetes.48.8.1593

[phy214285-bib-0059] Zurlo, F. , Larson, K. , Bogardus, C. , & Ravussin, E. (1990). Skeletal muscle metabolism is a major determinant of resting energy expenditure. The Journal of Clinical Investigation, 86, 1423–1427. 2243122 10.1172/JCI114857 PMC296885

